# Molecular characterization of genes encoding leucoanthocyanidin reductase involved in proanthocyanidin biosynthesis in apple

**DOI:** 10.3389/fpls.2015.00243

**Published:** 2015-04-10

**Authors:** Liao Liao, Sornkanok Vimolmangkang, Guochao Wei, Hui Zhou, Schuyler S. Korban, Yuepeng Han

**Affiliations:** ^1^Key Laboratory of Plant Germplasm Enhancement and Specialty Agriculture, Wuhan Botanical Garden of the Chinese Academy of SciencesWuhan, China; ^2^Department of Pharmacognosy and Pharmaceutical Botany, Faculty of Pharmaceutical Sciences, Chulalongkorn UniversityBangkok, Thailand; ^3^Graduate University of Chinese Academy of SciencesBeijing, China; ^4^Department of Biology, University of Massachusetts BostonBoston, MA, USA

**Keywords:** apple, anthocyanin, proanthocyanidin, leucoanthocyanidin reductase, anthocyanidin reductase

## Abstract

Proanthocyanidins (PAs) are the major component of phenolics in apple, but mechanisms involved in PA biosynthesis remain unclear. Here, the relationship between the PA biosynthesis and the expression of genes encoding leucoanthocyanidin reductase (LAR) and anthocyanidin reductase (ANR) was investigated in fruit skin of one apple cultivar and three crabapples. Transcript levels of *LAR1* and *ANR2* genes were significantly correlated with the contents of catechin and epicatechin, respectively, which suggests their active roles in PA synthesis. Surprisingly, transcript levels for both *LAR1* and *LAR2* genes were almost undetectable in two crabapples that accumulated both flavan-3-ols and PAs. This contradicts the previous finding that *LAR1* gene is a strong candidate regulating the accumulation of metabolites such as epicatechin and PAs in apple. Ectopic expression of apple *MdLAR1* gene in tobacco suppresses expression of the late genes in anthocyanin biosynthetic pathway, resulting in loss of anthocyanin in flowers. Interestingly, a decrease in PA biosynthesis was also observed in flowers of transgenic tobacco plants overexpressing the *MdLAR1* gene, which could be attributed to decreased expression of both the *NtANR1* and *NtANR2* genes. Our study not only confirms the *in vivo* function of apple *LAR1* gene, but it is also helpful for understanding the mechanism of PA biosynthesis.

## Introduction

Apple (*Malus* × *domestica* Borkh.), a member of the Rosaceae family, is one of the most widely cultivated fruit crops in the world. The apple is a diploid (2n = 34), with an autopolyploidy origin and a relatively small genome size of 750 Mb per haploid (Velasco et al., 2010). Apple fruits are rich in antioxidants such as proanthocyanidins and anthocyanins. Since anthocyanins play a critical role in fruit coloration, molecular mechanism underlying anthocyanin accumulation has recently been extensively studied in apple (Takos et al., 2006a; Ban et al., 2007; Espley et al., 2007; Li et al., 2012; Xie et al., 2012; Chagné et al., 2013; Dare et al., 2013; Vimolmangkang et al., 2013). In contrast, only a few studies have been conducted to investigate molecular basis of proanthocyanidin biosynthesis in apple (Chagné et al., 2012; Han et al., 2012; Henry-Kirk et al., 2012; Verdu et al., 2014).

Proanthocyanidins (PAs), also called condensed tannins, are phenolic polymers formed by condensation of flavan-3-ol monomeric units such as catechin and epicatechin, which are synthesized via a branch of anthocyanin biosynthesis pathway under the catalyzation of two enzymes, leucoanthocyanidin reductase (LAR) and anthocyanidin reductase (ANR). Namely, LAR catalyzes the conversion of leucoanthocyanidin (flavan-3,4-diol) to catechin, while ANR catalyzes the synthesis of epicatechin from anthocyanidin (Tanner et al., 2003; Xie et al., 2003, 2004). However, a more recent study indicates that ectopic expression of the tea *CsLAR* gene in tobacco results in the accumulation of higher level of epicatechin than that of catechin, suggesting that LAR may be also involved in the biosynthesis of epicatechin (Pang et al., 2013). Similarly, ANRs from grapevine and tea are proven to have epimerase activity and thus can convert anthocyanidin to a mixture of epicatechin and catechin (Gargouri et al., 2010; Pang et al., 2013). In cells, PAs are synthesized in the cytoplasm and accumulated into the vacuole. To date, the biosynthesis and accumulation of PAs have been reported in a variety of plant species (Matsui et al., 2004; Paolocci et al., 2007; Pang et al., 2008; Zhao and Dixon, 2009; Kitamura et al., 2010; Hammerbacher et al., 2014; Liu et al., 2014). However, many questions regarding the transport of PAs from cytosol to vacuoles and the polymerization of flavan-3-ol monomers are still open (Zhao et al., 2010).

PAs are very powerful antioxidants that can remove harmful free oxygen radicals from cells, and their antioxidant power is 20 times higher than that of vitamin C and 50 times higher than vitamin E (Shi et al., 2003). Since fruits are one of the main sources of PAs in our diets, many studies have been conducted to identify genes involved in PA biosynthesis and accumulation in fruit crops. Initially, structural genes of the PA-specific branch pathway, including *LAR* and *ANR*, were characterized in grapevine (Bogs et al., 2005; Pfeiffer et al., 2006; Maugé et al., 2010) and strawberry (Almeida et al., 2007). Subsequently, anthocyanidin synthase (ANS), which catalyzes the conversion of leucoanthocyanidin to anthocyanidin, is also proven to play an important role in the biosynthesis of PAs in apple fruit (Szankowski et al., 2009). However, increasing evidence shows PA accumulation is regulated at the transcriptional level by MYB TFs, including positive MYB regulators such as *VvMybPA1, VvMYB5b*, and *VvMybPA2* in grapevine (Bogs et al., 2007; Deluc et al., 2008; Terrier et al., 2009), *FaMYB9*/*FaMYB11* in strawberry (Schaart et al., 2013), and *DkMyb4* in persimmon (Akagi et al., 2009) and negative MYB regulators such as *VvMYBC2-L1* in grapevine (Huang et al., 2014). In addition, a basic leucine zipper transcription factor in persimmon, *DkbZIP5*, can bind to ABA-responsive elements in the promoter region of *DkMyb4* and thus induces the up-regulation of *DkMyb4* and the resultant PA biosynthesis (Akagi et al., 2012). In addition, two *MATE* (multidrug and toxic compound extrusion) genes, *VvMATE1* and *VvMATE2*, which is likely involved in transport of PAs from cytosol to vacuoles, are also reported in grapevine (Pérez-Díaz et al., 2014).

In apple, the predominant anthocyanin is cyanidin 3-galactoside (Tsao et al., 2003), which suggests that LAR and ANR enzymes act on leucocyanidin and cyanidin, respectively, to produce catechin and epicatechin (Figure S1). PAs account for up 80% of the total phenolic compounds in apple, and thus represent the predominant apple antioxidants (Wojdylo et al., 2008). More recently, a limited number of quantitative trait locus (QTL) mapping studies have been conducted to understand the genetic basis of PA accumulation in apple, and a major QTL together with several minor QTLs for the content of flavanol monomers and procyanidins and the average polymerization degree of procyanidins have been identified (Chagné et al., 2012; Khan et al., 2012; Verdu et al., 2014). A *LAR* gene within the major QTL interval is considered as a strong candidate controlling the accumulation of both flavanols and procyanidins (Chagné et al., 2012; Khan et al., 2012). However, an apple WD40-repeat gene, a homolog of *Arabidopsis* TRANSPARENT TESTA GLABRA1 (TTG1), was shown to activate the *AtBAN* promoter in cooperation with *Arabidopsis* TT2 and TT8 (Brueggemann et al., 2010). This finding suggests that PA accumulation in apple is probably regulated at the transcriptional level although no TFs in apple have to date been identified to be involved in regulation of PA biosynthesis.

We previously investigated the functionality of *ANR* gene family in apple, which is composed of one *MdANR1* gene on chromosome 10 and two allelic *MdANR2* genes (*MdANR2a* and *MdANR2b*) on chromosome 5 (Han et al., 2012). In this study, we further report on the role of *LAR* genes in PA biosynthesis in apple. Expression profiles of both *LAR* and *ANR* genes were investigated in cultivated and wild apple fruits, and functional characterization was conducted for an apple *LAR1* gene via ectopic expression in tobacco. Our study indicates that the apple *LAR1* and *ANR2* genes probably play an important role in the biosynthesis of catechin and epicatechin, respectively, and ectopic expression of apple *LAR1* genes in tobacco causes a significant decrease in both anthocyanin and PA accumulation in flowers. This finding is not only helpful for understanding the mechanism of the PA biosynthesis, but it will also aid in future attempts to manipulate PA biosynthesis in apple as well as in other plants.

## Materials and methods

### Plant material

Apple fruits at enlargement and mature stages were collected. To facilitate description, fruit at enlargement stage was referred to as immature fruit (hereinafter the same). Apple accessions were Aihuahong (*Malus asiatica* Nakai var. nana Li.), Xijinhaitang [*Malus sikkihensis* (wenzig) Koehne ex Schneid.], Xiongyuehaitang [*Malus prunifolia* (Willd.) Borkh.], and Fuji (*Malus domestica* Borkh.). The maturity of the fruits was assessed by checking the color of the peel and a confirmation of the seed color changing to brown. Each accession had three replicates, consisting of five fruits. Fruit skins were peeled off and used for the studies. *Nicotiana tabacum* cv. Petite Havana SR1 was selected for gene functional study. Tobacco flowers at full-bloom stage were harvested for gene expression analysis and chemical analysis.

### Quantitative PCR for gene expression analysis

Total RNA was extracted using ZP401 kit (Beijing Zoman Biotechnology Co., Ltd., Beijing, China) following the manufacture's protocol. Total RNR were then treated with DNase I (Takara, Dalian, China) to remove any contamination of genomic DNA. Approximately 2 μg of total RNA was used for cDNA synthesis using PrimeScript™ RT-PCR Kit (Takara).

The qPCRs were performed in 96-well plates using a 7500 Real Time PCR System (Applied Biosystems). All analyses were repeated three times using biological replicates. The SYBR Green real-time PCR assay was carried out in a total volume of 20 μL reaction mixture containing 10 μL of 2 × SYBR Green I Master Mix (Takara, Dalian, China), 0.2 μM of each primer, and 100 ng of template cDNA. An apple actin gene (GenBank accession no. CN938023) and a tobacco actin gene (GenBank accession no. AY179605) were used as a constitutive control. The amplification program consisted of 1 cycle of 95°C for 3 min, followed by 40 cycles of 95°C for 30 s and 60°C for 30 s. Melting curve analysis was performed at the end of 40 cycles to ensure the proper amplification of target fragments. Fluorescence readings were consecutively collected during the melting process from 60 to 90°C at the heating rate of 0.5°C/s. Primer sequences used for real-time PCR analysis were listed in Tables S1.

### Expression vector construction and tobacco transformation

A pair of primers (5′-TGACGAGCTCATGACCGTTTCATCTTCTCTTTCTG-3′/5′-ATACGGATCCTCAAGCACAAGTGGCAGTGACAG-3′) was designed to amplify the full coding sequences of the *MdLAR1* gene using cDNA from fruits of cv. Fuji as templates. The forward and reverse primers contain *Sac*I/*Bam*HI sites at the 5′ end, respectively. The PCR amplification was conducted using proofreading DNA polymerase *Pfu* (Takara, Dalian, China), and PCR products, digested with *Bam*HI and *Sac*I, were ligated into *Sac*I/*Bam*HI-digested pCAMBIA1301s binary vector. The gene construct was introduced into *Agrobacterium tumefaciens* strain GV3101 by electroporation. *Agrobacterium*-mediated transformation in tobacco was conducted according to our previously reported protocol (Han et al., 2012).

### Measurement of proanthocyanidin and anthocyanin contents

Tissue samples, tobacco flowers or apple skins, were ground to fine power and then subjected to analysis of proanthocyanidin and anthocyanin contents. Soluble PAs were extracted and quantified using the DMACA-HCl Protocol (Li et al., 1996). Anthocyanin content was assayed following our previously reported protocol (Zhou et al., 2014), with some modification. Briefly, approximately 0.1 g of finely-ground tissues was extracted twice with 1 ml extraction solution (0.1% HCl in methanol) and the supernatants were combined and diluted to 3-mL final volume. Then, 200 μL supernatant was mixed with 2.8 ml of buffer A (0.4 M KCl, adjusted to pH 1.0 with HCl) or buffer B (1.2 N citric acid, adjusted to pH 4.5 with NaH_2_PO_4_ and NaOH). Absorbance of the mixture was measured at 510 and 700 nm. The anthocyanin content was calculated using the following formula: TA = A × MW × 15 × 1000 × V/e, where TA stands for total anthocyanin content as cyanidin-3-O-glucose equivalent (mg/100 g), V for final volume (ml), and A = [(A_510_ – A_700_) at pH1.0] – [(A_510_ – A_700_) at pH 4.5], e is absorbance of cyanindin-3-glucoside (26,900), MW is molecular weight of cyanindin-3-glucoside (449.2). All analyses were repeated three times using biological replicates.

### LC-MS/MS analysis of flavan-3-ol monomers

PAs were extracted from finely-ground tissues of tobacco flowers and apple skins according to our previously reported protocol (Han et al., 2012), with some modification. Briefly, the tissue was soaked in 1 mL of 70% (v/v) acetone containing 0.1% (w/v) ascorbate, and incubated for 24 h in darkness. The extract was centrifuged and the supernatant was transferred to a new 1.5 ml microcentrifuge tube. The extract was partially purified by adding equal amount of chloroform and the supernatant was collected. The solvent was evaporated, and the extract was resuspended in 500 μL of water/methanol (1:1, v/v).

PAs were identified using liquid chromatography–tandem mass spectrometry (LC-MS/MS) and their contents were calculated by comparison with commercial standards, including catechin and epicatechin (Sigma). The ESI-MS/MS system (ThermoFisher Scientific, Pittsburgh, PA) equipped with a Thermo Scientific Accela 1250 HPLC was used. The HPLC Separation was performed on a Hisep C18-T column (5 μm, 4.6 × 150 mm; Weltech Co., Ltd., Wuhan, China). HPLC mobile phase consisted of A (0.2% acetonitrile in H_2_O) and B (Methanol) and the flow rate was set at 1.2 mL min^−1^. The gradient for catechin and epicatechin was as follows: 0 min, 100% A; 12 min, 50% A; 13–20 min, 50% A; and 21–30 min, 100% A. The injection volumes were 20 μL for samples and 10 μL for PA standards. The PAs were observed under UV detector at 280 nm and determined according to retention time of standards (Figure S2). Mass spectra were acquired in positive ion mode and multiple reaction monitoring was used to identify and quantify catechin and epicatechin (m/z 291.0/139.2/123.1). All analyses were repeated three times using biological replicates.

## Results

### Flavonoid content in wild and cultivated apples

**Figure 1:**
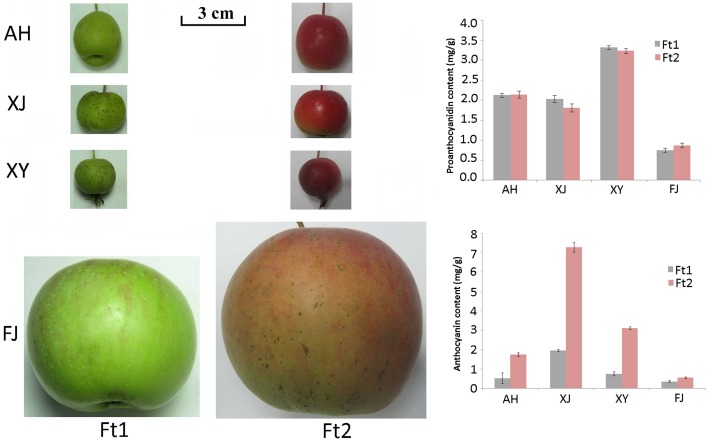
**Fruit skin color of four *Malus* accessions and the proanthocyanidin and anthocyanin content in the skin**. Fruits were collected at enlargement (Ft1) and mature (Ft2) stages. Abbreviations for *Malus* accessions are as follows: AH, Aihuahong; XJ, Xijinhaitang; XY, Xiongyuehaitang; FJ, Fuji.

Flavonoid content was investigated in four *Malus* accessions, including an apple cultivar Fuji (FJ) and three crabapples, Aihuahong (AH), Xijinhaitang (XJ), and Xiongyuehaitang (XY). The mature fruit skin of all the four apple accessions showed a significant increase in anthocyanin content when compared with immature fruit skin (*P* < 0.025, Figure 1). Anthocyanin content ranged from 0.56 to 7.28 mg/g in mature fruit skin, and from 0.36 to 1.97 mg/g in immature fruit skin. The crabapple XJ had the highest level of anthocyanins in both immature and mature fruit skins, followed by XY, AH, and FJ accessions. In contrast, the PA content showed no significant change in fruit skin between enlargement and mature stages (*P* > 0.05). The PA content ranged from 0.74 to 3.31 mg/g in immature fruit skin, and from 0.87 to 3.23 mg/g in mature fruit skin. The crabapple XY and the cultivar FJ had the highest and lowest levels of the PAs in fruit skins, respectively.

**Figure 2:**
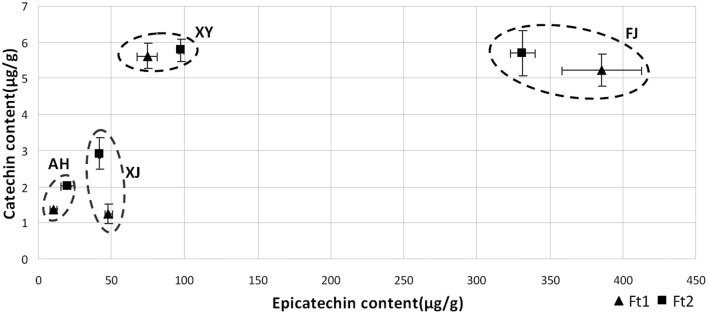
**Flavan-3-ol content in fruit skin of four *Malus* accessions at fruit enlargement and mature stages**. The amounts of catechin and epicatechin were determined by LC-MS/MS against the standard curve.

For flavan-3-ols, fruit skin of all the four apple accessions contained both catechin and epicatechin, with epicatechin being predominant (Figure 2). Epicatechin content ranged from 10.19 to 385.50 μg/g in immature fruit skin, and from 19.98 to 331.38 μg/g in mature fruit skin. The crabapple XJ had the highest level of epicatechin content in both immature and mature fruit skins, followed by FJ, XJ, and AH accessions. Catechin content ranged from 1.24 to 5.62 μg/g in immature fruit skin, and from 2.01 to 5.77 μg/g in mature fruit skin. The crabapple XY and cv. FJ had the highest level of catechin content in mature fruit skin, followed by XJ and AH accessions. Overall, all the four *Malus* accessions showed a great variation in the flavan-3-ol and PA content.

### Expression profile of PA biosynthesis genes in apple

**Figure 3:**
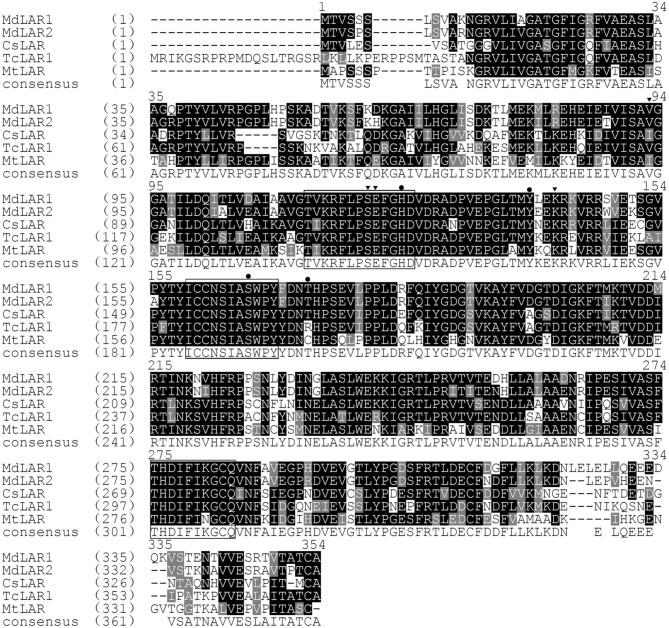
**Alignment of deduced amino acid sequences of the two apple *LAR* genes and their three homologs, including *Camellia sinensis CsLAR* (ADZ58167), *Theobroma cacao TcLAR1* (XP_007046315), *Medicago truncatula MtLAR* (XP_003591830)**. Conserved amino acid sequences are indicated by a black ground and similar amino acids by a light gray background. The RFLP, ICCN, and THD motifs are boxed. Amino acids interacting with NADPH in the active site are highlighted in a black colored triangle and amino acids interacting with leucoanthocyanidin are highlighted in a black colored dot. The numbering of amino acids follows the scheme for MdLAR1 and MdLAR2.

**Figure 4:**
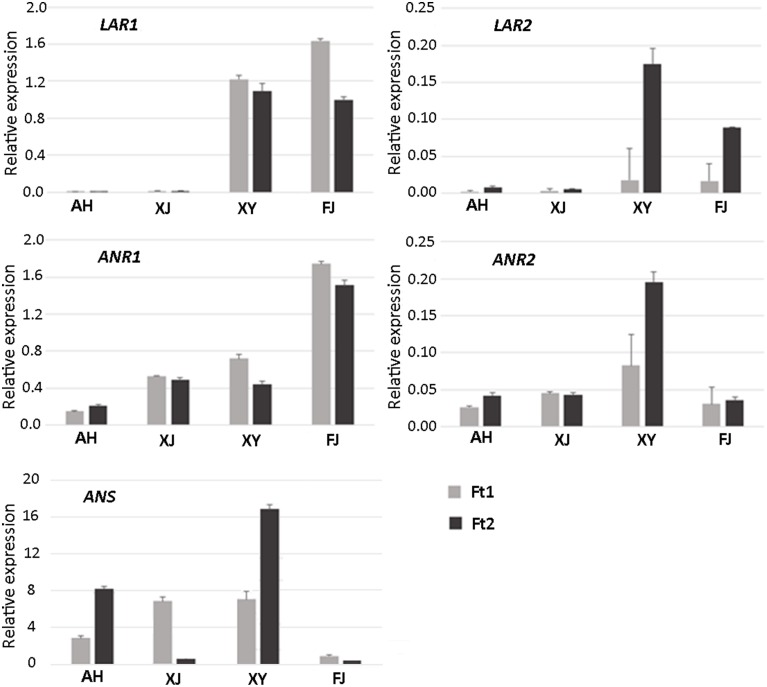
**Expression profiles of PA pathway biosynthetic genes in fruit skin of four *Malus* accessions**.

Genes encoding LAR, ANR, and ANS are closely related to PA biosynthesis (Figure S1). Two apple *LAR* genes, designated *MdLAR1* and *MdLAR2*, have been reported in previous study (Takos et al., 2006b). Comparison of their DNA sequences with the apple reference genome (Velasco et al., 2010) revealed that *MdLAR1* and *MdLAR2* are located on linkage groups (LG) 16 and 13, respectively. *MdLAR1* and *MdLAR2* share 93 and 91% identity in coding DNA and amino acid sequences, respectively, and the RFLP, ICCN, and THD motifs are identical between MdLAR1 and MdLAR2 proteins (Figure 3). Likewise, we previously identified two *ANR* genes, termed *MdANR1* and *MdANR2*, in the apple genome (Han et al., 2012). However, only one copy of the *ANS* gene is present in the apple genome (Velasco et al., 2010). Thus, the expression profile of these five PA biosynthesis genes was investigated in the fruit skin of the four *Malus* accessions as mentioned above and the result is shown in Figure 4.

*LAR1* was highly expressed in immature and mature fruit skins of cv. FJ and the crabapple XY, while its transcript level was almost undetectable in immature and mature fruit skins of the two crabapples AH and XJ. *LAR2* was highly expressed in mature fruit skin of cv. FJ and the crabapple XY, but weakly expressed in immature fruit skin. Transcript accumulation of *LAR2* was almost undetectable or extremely low in immature and mature fruit skins of the two crabapples AH and XJ. Both *ANR1* and *ANR2* were expressed in immature and mature fruit skins of all the tested accessions. Transcript accumulation of *ANR1* showed the highest level in fruit skin of cv. FJ, whereas, transcript accumulation of *ANR2* showed the highest level in fruit skin of the crabapple XY. To confirm the reliability of gene expression profiling result, the qRT-PCR products were cloned and sequenced. The DNA fragments of both *LAR1* and *LAR2* are identical among the four *Malus* accessions (Figure S3). In contrast, three single nucleotide polymorphisms were detected for the DNA fragments both *ANR1* and *ANR2*, but no DNA polymorphism at the primer binding sites. In addition, the *ANS* gene was expressed in immature and mature fruit skins of all the three crabapples, but its transcript level was extremely low in mature fruit skin of the wild accession XJ. In contrast, transcript accumulation of *ANS* gene was extremely lower in mature and immature fruit skins of cv. FJ.

### Relationship between the PA biosynthesis gene expression and the flavan-3-ol and PA accumulation

**Figure 5:**
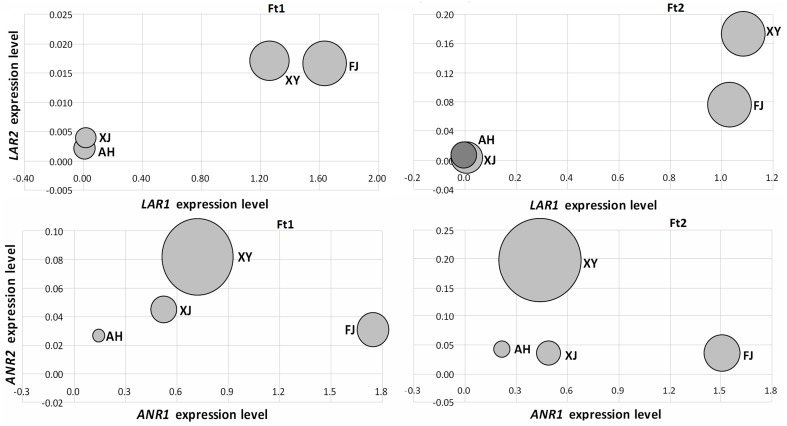
**Relationship between PA biosynthetic gene expression levels and the amount of flavan-3-ol content in fruit skins of four apple accessions**. The size of bubbles represents the amount of PA contents either catechin or epicatechin. The amount of catechin was used to compare with *MdLAR1* and *MdLAR2* expression levels, whereas, the amount of epicatechin was used to compare with *MdANR1* and *MdANR2* expression levels.

We initially investigated the relationship between the expression levels of *LAR* and *ANR* genes and the concentrations of catechin and epicatechin, respectively, in fruit skins of four *Malus* accessions (Figure 5). The *LAR1* expression level was significantly correlated with the catechin content in both immature and mature fruit skins, with the Spearman correlation coefficients (*r*) 0.99 (*P* < 0.05) and 0.98 (*P* < 0.05), respectively. Likewise, the *ANR2* expression level was significantly correlated with the epicatechin content in both immature (*r* = 0.95, *P* < 0.05) and mature (*r* = 0.96, *P* < 0.05) fruit skins. However, the expression level of *LAR2* or *ANR1* showed no significant correlation with the content of catechin or epicatechin, respectively, in both immature and mature fruit skins. Subsequently, we investigated the relationship between the expression level of *LAR* and *ANR* genes and the PA concentration (Figure S4). The highest level of correlation was observed between the expression level of *ANR2* and the PA concentration in immature (*r* = 0.80, *P* > 0.05) and mature (*r* = 0.87, *P* > 0.05) fruit skins, but the Spearman correlation coefficients did not reach statistical significance. This indicates that there is no significant correlation between the expression level of both *LAR* and *ANR* and the PA concentration in both immature and mature fruit skins.

### Ectopic expression of *MdLAR1* in tobacco

**Figure 6:**
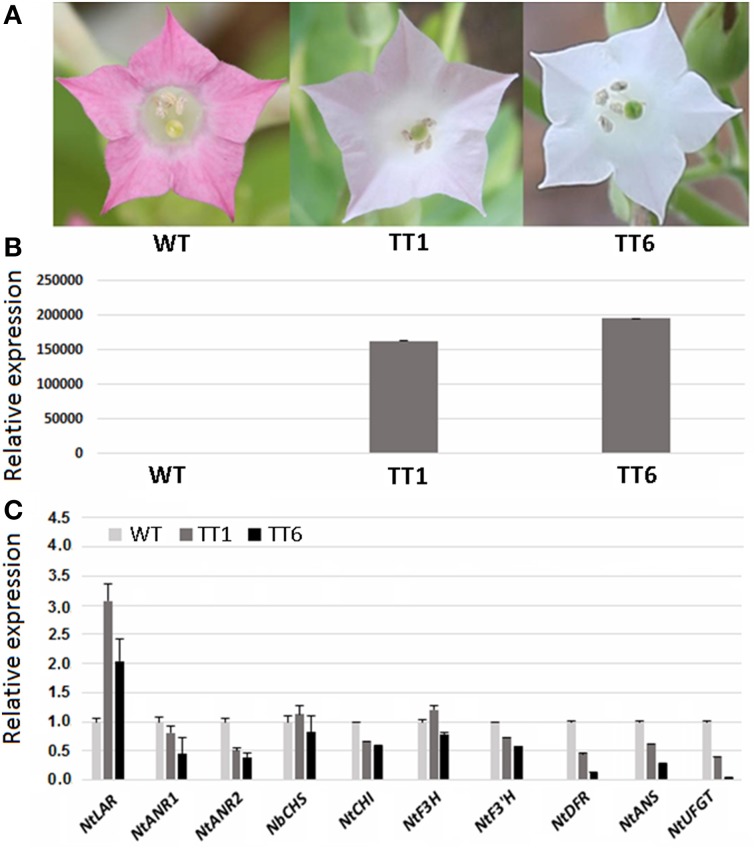
**Ectopic expression of *MdLAR1* gene in tobacco. (A)** Flower color of transgenic tobacco (TTI, transgenic tobacco type I; TTII, transgenic tobacco type II) and wild-type tobacco (WT). **(B)** Gene expression level of *MdLAR1* in transgenic flowers. **(C)** Expression profiles of flavonoid-related biosynthetic genes in transgenic flowers relative to wild-type tobacco flowers.

As mentioned above, *MdLAR1* and *MdLAR2* share high level identity in both coding DNA and amino acid sequences and the conserved LAR motifs, RFLP, ICCN, and THD, are identical between MdLAR1 and MdLAR2 proteins. Moreover, *MdLAR1* is located in the major QTL interval controlling the accumulation of flavanols and procyanidins (Chagné et al., 2012; Khan et al., 2012). Thus, only the *MdLAR1* gene was selected for functional analysis. The coding region of *MdLAR1* was transferred into tobacco under control of the *Cauliflower mosaic virus* (CaMV) 35S promoter, and eight T_0_ transgenic lines were generated. During the vegetative growth stage, these transgenic plants were indistinguishable from wild-type plants. During the flowering stage, however, flower colors of two transgenic lines, TT1 and TT6, were different from those of wild-type plants. Wild-type plants bore red flowers, whereas, TT1 and TT6 produced pale pink-colored and pure white flowers, respectively (Figure 6A). Thus, these two transgenic lines were selected and subjected to analysis of gene expression and flavonoid content.

The qRT-PCR analysis revealed that the *MdLAR1* gene showed extremely high levels of expression in flowers of both TT1 and TT6 transgenic lines (Figure 6B). Pale-pink and white flowers of the transgenic lines accumulated certain amounts of anthocyanin, but these levels were significantly lower than those of wild-type flowers (Table 1). Surprisingly, the PA contents in either white- or pale pink-colored transgenic flowers were also significantly lower than that of wild-type flowers. In contrast, both pale-pink and white flowers of the transgenic lines accumulated slightly higher levels of epicatechin than did wild-type flowers, but the changes did not reach statistical significance. Likewise, no significant change in catechin content was observed between wild-type flowers and either white- or pale pink-colored transgenic flowers. Taken together, ectopic expression of the *MdLAR1* gene in tobacco inhibited the biosynthesis of both anthocyanins and PAs in flowers, but exhibited no effect on flavan-3-ol accumulation.

**Table 1:** HPLC analysis of flavonoid in wild-type and transgenic tobacco flowers^*^.

**Tobacco**	**Anthocyanin (mg/g)**	**Proanthocyanidin (mg/g)**	**Epicatechin (μg/g)**	**Catechin (μg/g)**
WT	3.16 ± 0.07^a^	0.76 ± 0.05^a^	2.33 ± 0.51^a^	3.67 ± 0.75^a^
TT1	0.62 ± 0.05^b^	0.56 ± 0.04^b^	2.47 ± 0.14^b^	4.49 ± 0.65^b^
TT6	0.51 ± 0.07^b^	0.56 ± 0.03^b^	2.36 ± 0.23^a^	4.33 ± 0.82^a^

* *All data correspond to mean values of three biological replicates. Values with different letters (a and b) within the same column are significantly different at the 0.05 level of probability*.

qRT-PCR analysis was also conducted to investigate the coordinate interaction of the *MdLAR1* gene with other flavonoid pathway genes in transgenic tobacco flowers, including *NtCHS, NtCHI, NtF3H, NtF3*'*H, NtDFR, NtANS, NtUFGT, NtLAR, NtANR1*, and *NtANR2*. Overexpression of the *MdLAR1* gene in tobacco greatly influenced expression of flavonoid structural genes in flowers (Figure 6C). For example, expression of *NtCHI, NtF3*'*H, NtDFR, NtANS, NtUFGT, NtANR1*, and *NtANR2*, was down-regulated in flowers of both TT1 and TT6 transgenic lines, while expression of *NtLAR* was up-regulated in flowers of both transgenic lines. Of all genes investigated, the two genes *NtUFGT* and *NtDFR* showed extremely low levels of expression in white-colored transgenic flowers.

## Discussion

Apple fruits accumulate usually high levels of PAs, which contributes to human health and organoleptic property (Renard et al., 2007). However, mechanisms involved in the biosynthesis of PAs remain unclear. We here describe the functional analysis of a *LAR* gene in apple. The LAR enzyme is known to compete with the ANS enzyme to convert leucoanthocyanidin into catechin. The *Arabidopsis* genome does not contain an *LAR* ortholog, and thus catechin is not detected in the seed coat (Abrahams et al., 2003; Tanner et al., 2003). In this study, transcript accumulation of both *LAR1* and *LAR2* genes are almost undetectable in fruit skin of two wild apple species AH and XJ, which is consistent with the low concentration of catechin in fruit peel. In contrast, *LAR1* and *LAR2* genes are expressed in fruit skin of the crabapple XY and cv. FJ. However, the concentration of catechin is extremely lower than that of epicatechin, with epicatechin being the predominant flavan-3-ol monomer in fruit skin. This inconsistency has also been reported in *Medicago*, in which *LAR* is expressed, but the PAs are composed almost entirely of epicatechin units (Pang et al., 2007). In the wild apple *Malus sieversii*, silencing *ANS* gene results in an increase in *LAR* transcript level, together with a decrease in *ANR* transcript level (Szankowski et al., 2009). Interestingly, an increase in epicatechin accumulation is observed in the *ANS*-silenced apples. This increase suggests the possibility of an alternative biosynthetic pathway to epicatechin such as epimerization of catechin to epicatechin and depolymerization in a non-stereospecific manner from polymeric epicatechin derivatives (Szankowski et al., 2009). Further studies are needed to address whether the alternative biosynthetic pathway to epicatechin is also responsible for the finding that the flavan-3-ols are composed almost entirely of epicatechin in fruit skin of the four *Malus* accessions tested in this study.

The mature fruit skin of the apple cultivar FJ shows a slight increase in PA accumulation when compared with the immature fruit skin. In contrast, three crabapples accumulate slightly lower levels of PAs in the skin of mature fruit than in the skin of immature fruit. The fruit size of the cultivar FJ shows a significant increase in mature stage, but no obvious change for the three crabapples. Therefore, it seems that fruit enlargement has little impact on PA accumulation in apple.

There are two copies of both *ANR* and *LAR* genes in the apple genome. *MdANR1* and *MdANR2* are located on homologous chromosome pairs 10 and 5, respectively (Han et al., 2012). Similarly, *MdLAR1* and *MdLAR2* are located on homologous chromosome pairs 16 and 13, respectively. Thus, duplication of both *ANR* and *LAR* genes in apple is attributed to the polyploidy origin of the apple genome (Velasco et al., 2010; Han et al., 2012). For the two *LAR* genes in apple, only *LAR1* shows a significant correlation between its transcript level and the catechin content. Likewise, transcript level of the apple *ANR2* is significantly correlated with the epicatechin content, but not for the apple *ANR1*. Thus, the *LAR1* and *ANR2* genes are likely crucial for the biosynthesis of catechin and epicatechin in apple peel, respectively. This in turn suggests that functional divergence between the two apple duplicated genes encoding both LAR and ANR may occur during the course of evolutionary development of apple.

Genetic mapping studies indicate that the *LAR1* gene at the mQTL hotspot on LG16 is considered as a strong candidate regulating the accumulation of metabolites such as catechin, epicatechin, and procyanidins in apple (Chagné et al., 2012; Khan et al., 2012). However, our result indicates that *LAR1* transcript level does not show any significant correlation with either the epicatechin content or the PA content. In the two crabapples AH and XJ, transcript accumulation of *LAR1* is almost undetectable, whereas both epicatechin and PAs are accumulated in fruit peel. Moreover, the *LAR1* gene is highly expressed in fruit skin of cv. FJ. However, the PA content in fruit skin of cv. FJ is significantly lower than those in fruit skin of the two crabapples AH and XJ. All these results suggest that the apple *LAR1* gene is unlikely responsible for the epicatechin and PA biosynthesis. Besides the *LAR1* gene, several transcription factor genes encoding MYB, bHLH, AP2, and bZIP proteins are also located in the mQTL hotspot. It is worthy of further study to address whether these transcriptional factors are involved in the regulation of the flavanol and PA biosynthesis in apple. In addition, the *ANS* gene also plays an important role in PA biosynthesis as its silencing causes a decrease in PA biosynthesis in *Malus sieversii* (Szankowski et al., 2009). Transcript level of *ANS* is extremely low in fruit skin of cv. FJ. It is unclear whether or not the PA accumulation at low level in cv. FJ could be partially attributed to the extremely low expression of the *ANS* gene.

Our previous study indicates that ectopic expression of *MdANR* genes in tobacco inhibits expression of both *CHI* and *DFR* genes in flowers, resulting in a decrease in anthocyanin accumulation (Han et al., 2012). Similar result is also observed for the *MdLAR1* gene in this study. Overexpression of *MdLAR1* suppresses expression of anthocyanin pathway genes in flowers, including *CHI, F3'H, DFR, ANS*, and *UFGT*, leading to a significant loss of anthocyanin. Thus, it is clear that loss of color in transgenic tobacco flowers may be due to inhibition of expression of the late genes in anthocyanin biosynthetic pathway. Decreased expression of all the late anthocyanin biosynthetic genes also suggests that pathway flux tends to be shifted away from anthocyanin toward PAs. Like transgenic tobacco plants overexpressing *MdANR* genes, transgenic tobacco lines overexpressing the *MdLAR1* gene accumulate slightly higher levels of catechin and epicatechin in flowers when compared with wild-type plants. What is unexpected is a decrease in the PA content in flowers of transgenic tobacco plants overexpressing the *MdLAR1* gene. This is somewhat consistent with the finding that introduction of the tea *LAR* gene in the *PAP1*-expressing tobacco does not increase the soluble PA accumulation (Pang et al., 2013).

Ectopic expression of *MdANR* genes in tobacco suppresses expression of *NtLAR*, and increases expression of *NtANR1, NtANR2*, and *NtANS* (Han et al., 2012). In turn, ectopic expression of *MdLAR1* in tobacco suppresses expression of *NtANR1, NtANR2*, and *NtANS*, and increases expression of *NtLAR*. Flowers of transgenic tobacco lines overexpressing the *MdLAR1* gene accumulate higher levels of catechin than of epicatechin, whereas, flowers of transgenic tobacco lines overexpressing the *MdANR* genes accumulate higher levels of epicatechin than of catechin (Han et al., 2012). These results indicate the transcription of *LAR, ANR*, and *ANS* might be regulated by the feedback mechanism (Tanner et al., 2003; Liu et al., 2013). In other words, high concentrations of catechin stimulate the *LAR* expression, whilst high concentrations of epicatechin stimulate the *ANS* and *ANR* expression. This is similar to a previous report in which high concentrations of trans-p-coumaric acid can stimulate the *CHS* expression (Loake et al., 1991). In addition, there is a potential competition between LAR and ANR enzymes as reported in our previous study (Han et al., 2012). Both LAR and ANR enzymes are NAPDH-dependent reductase and overexpression of one of them will offer little opportunity for another to accept NAPDH. This may also be partially responsible for the observed mutual inhibition between the *ANR* and *LAR* expression. Taken together, the PA biosynthesis is likely co-regulated by structural genes such as *LAR* and *ANR*, and the mutual inhibition between the *ANR* and *LAR* expression may cause the decrease in PA accumulation in flowers of transgenic tobacco plants overexpressing the *MdLAR1* gene.

### Conflict of interest statement

The authors declare that the research was conducted in the absence of any commercial or financial relationships that could be construed as a potential conflict of interest.
