# Supplementary material for: Molecular characterization of genes encoding leucoanthocyanidin reductase involved in proanthocyanidin biosynthesis in apple
Source: Front Plant Sci. 2015 Apr 10;6:243. doi: 10.3389/fpls.2015.00243 (PMC4392590; doi:10.3389/fpls.2015.00243)
Supplement: Supplementary file 1 [file Table1.DOC]

**Table S1** Primers used for qRT-PCR analysis of genes involved in flavonoid biosynthesis in apple and tobacco

| Species | Gene | Forward primer(5'→3') | Reverse primer(5'→3') | Accession no. |
| --- | --- | --- | --- | --- |
| Apple | *MdLAR1* | CGGTGTTTTCGGTGGAGACT | TTCAGGACCTTGGATGAGTGC | AY830131 |
|  | *MdLAR2* | CATCACTGAAAATCACTTACTAGCCC | CAAGGTCCTGAAAGAATCTCCG | AY830132 |
|  | *MdANR1* | GCAGAAGTAGAAAAACCAAGTCTCA | TTACGGTAAGCCAGACAATAGAGAG | JN035299 |
|  | *MdANR2* | CATGTGGAGGATGTCTGCCG | GTATCTTTTGTTGAGGAACCGAGC | JN035300 |
|  | *MdANS* | GAGGCTGGAGAAAGAAGTTGGTGGA | ATGTTGTGGAGGATGAAGGTGAGTG | AF117269 |
|  | *MdUFGT* | AGGAGCAGATGGCAATAGCG | CACGAAGGCTCCGACCG | AF117267 |
|  | *MdActin* | TGACCGAATGAGCAAGGAAATTACT | TACTCAGCTTTGGCAATCCACATC | CN938023 |
| Tobacco | *NtActin* | TAGGGATGTGAAGGAGAAGTTGG | TTCTGGGCAGCGGAATCTC | AY179605 |
|  | *NtLAR1* | TTCAGGACCTTGGATGAGTGC | ACGGTGTTTTCGGTGGAGAC | JX892957 |
|  | *NtANR1* | CATTTGACTTTCCCAAACGC | ATTGGGCTTTTGAGTTGTGC | AM791704 |
|  | *NtANR2* | TGTTCCCACTTGGGATGATA | TGCACCTATACTCTGTTAGTGGC | DW003895 |
|  | *NbCHS* | AGAAAAGCCTTGTGGAAGCA | ACTTGGTCCAAAATTGCAGG | EF421432 |
|  | *NtCHI* | GAAATCCTCCGATCCAGTGA | CAACGTTGACAACATCAGGC | AB213651 |
|  | *NtF3H* | ACAGGGTGAAGTGGTCCAAG | CCTTGGTTAAGGCCTCCTTC | AB289450 |
|  | *NtF3’H* | TCCAAGAATACTGGCCCAAG | CTCACAACTCTCGGATGCAA | AB289449 |
|  | *NtDFR* | CAGTTGCTTCCCTTTTCTACCC | TTGACTTTCCTGTTCCATTTGC | EF421431 |
|  | *NtANS* | AGTGGGTAACGGCAAAGTGTG | CCCTCTGTGAAGAATGCTTTTGT | AB289447 |
|  | *NtUFGT* | AATGGCACTACCCCTATTGGAC | ACCCCTGTTTCCTCCTCTGC | GQ395697 |
