# Supplementary figures and images for: Molecular characterization of genes encoding leucoanthocyanidin reductase involved in proanthocyanidin biosynthesis in apple
Source: Front Plant Sci. 2015 Apr 10;6:243. doi: 10.3389/fpls.2015.00243 (PMC4392590; doi:10.3389/fpls.2015.00243)

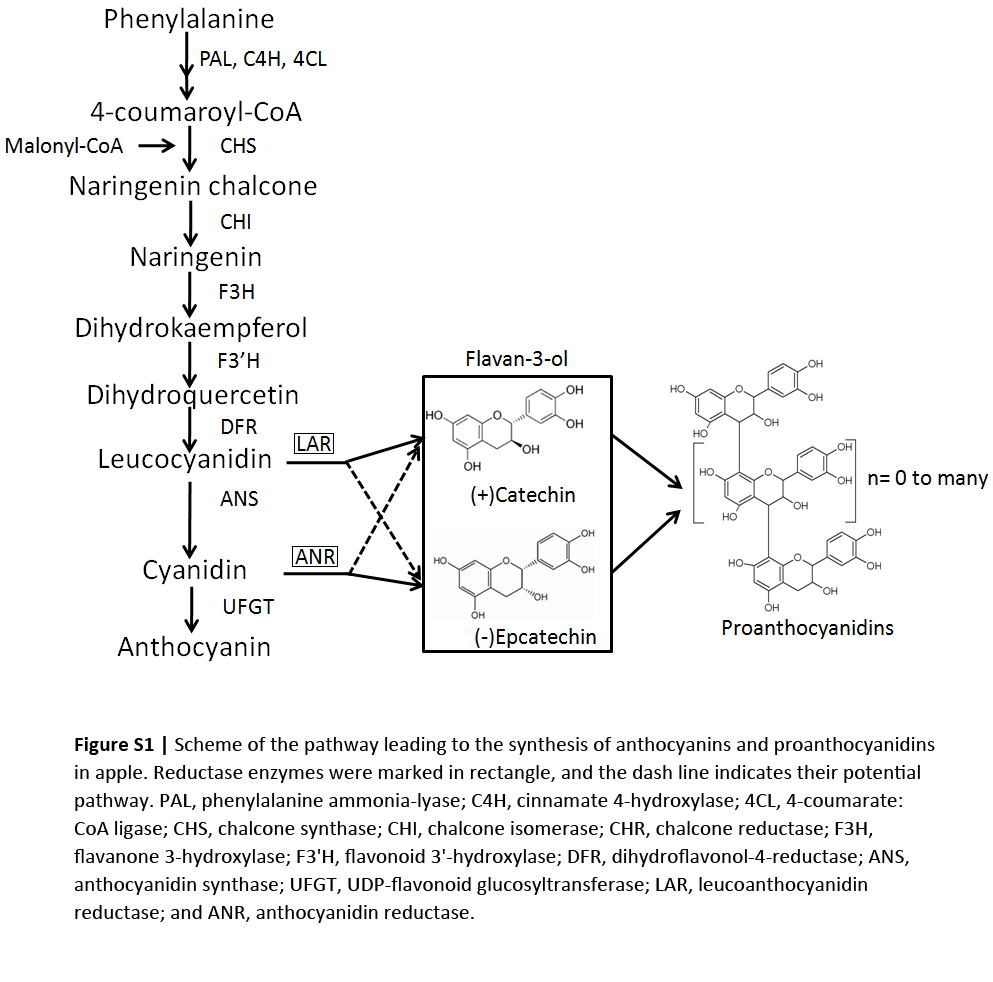

Supplement: Supplementary file 2 [file Image1.TIF]

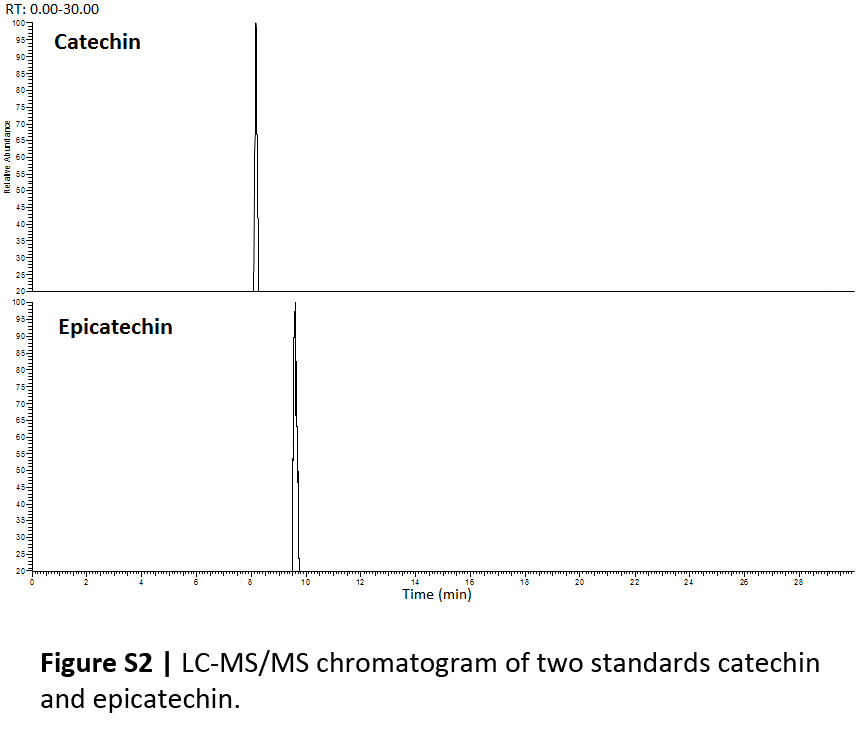

Supplement: Supplementary file 3 [file Image2.TIF]

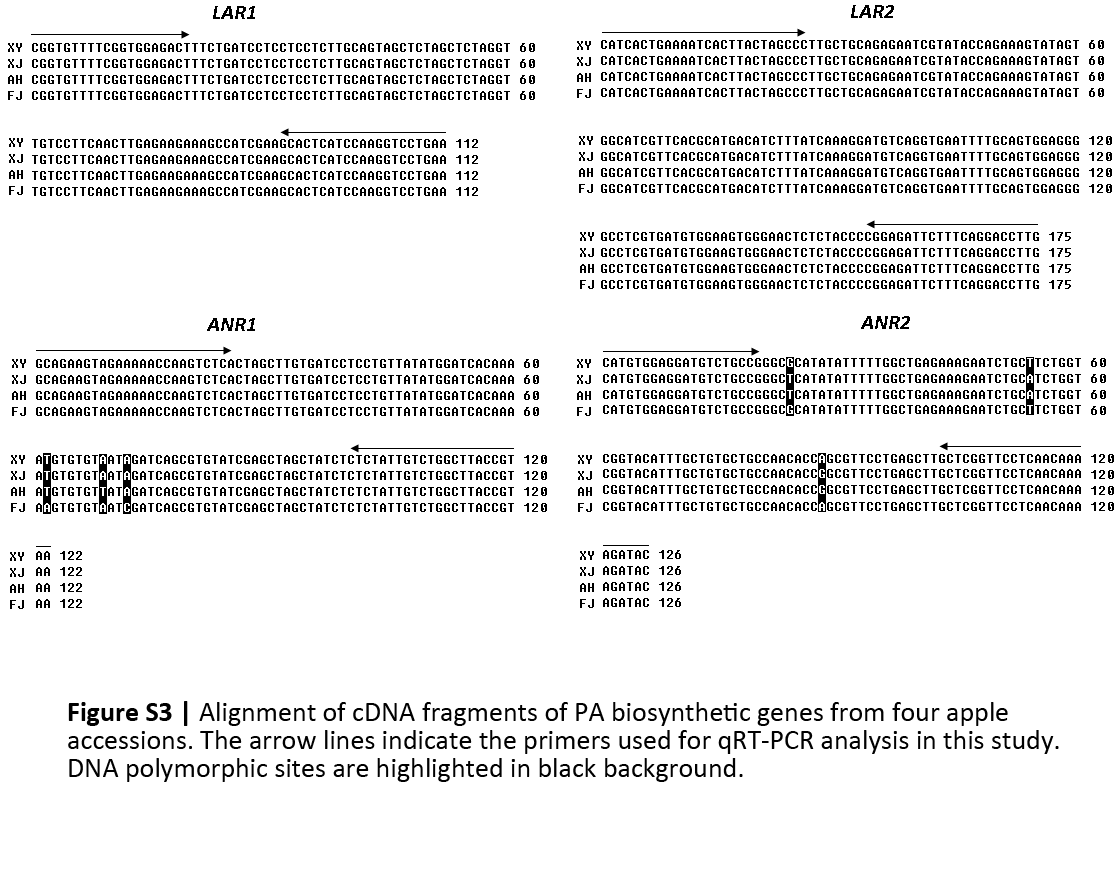

Supplement: Supplementary file 4 [file Image3.TIF]

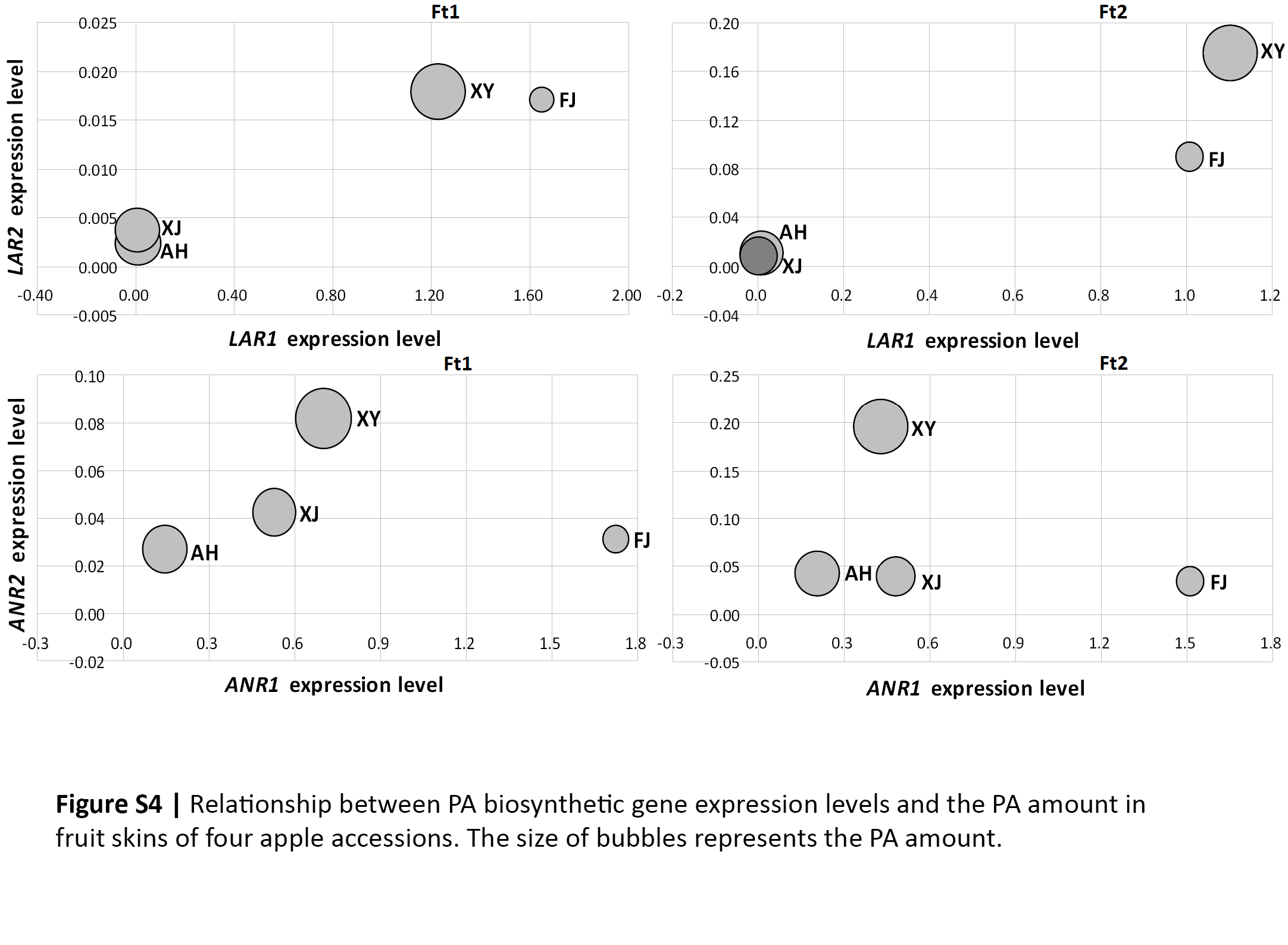

Supplement: Supplementary file 5 [file Image4.TIF]
